# Multiparameter Analysis, including EMT Markers, on Negatively Enriched Blood Samples from Patients with Squamous Cell Carcinoma of the Head and Neck

**DOI:** 10.1371/journal.pone.0042048

**Published:** 2012-07-26

**Authors:** Priya Balasubramanian, James C. Lang, Kris R. Jatana, Brandon Miller, Enver Ozer, Mathew Old, David E. Schuller, Amit Agrawal, Theodoros N. Teknos, Thomas A. Summers, Maryam B. Lustberg, Maciej Zborowski, Jeffrey J. Chalmers

**Affiliations:** 1 William G. Lowrie Department of Chemical and Biomolecular Engineering, The Ohio State University, Columbus, Ohio, United States of America; 2 Department of Otolaryngology – Head and Neck Surgery, The Ohio State University, Columbus, Ohio, United States of America; 3 Department of Otolaryngology-Head and Neck Surgery, Nationwide Children's Hospital, Columbus, Ohio, United States of America; 4 Department of Pathology and Laboratory Services, Walter Reed National Military Medical Center, Bethesda, Maryland, United States of America; 5 Department of Internal Medicine, Breast Medical Oncology, James Cancer Hospital and Ohio State University Comprehensive Cancer Center, Columbus, Ohio, United States of America; 6 Department of Biomedical Engineering, Cleveland Clinic, Cleveland, Ohio, United States of America; 7 Analytical Cytometry Shared Resource, OSU Comprehensive Cancer Center, Columbus, Ohio, United States of America; Virginia Commonwealth University, United States of America

## Abstract

Epithelial to mesenchymal transition (EMT) has been hypothesized as a mechanism by which cells change phenotype during carcinogenesis, as well as tumor metastasis. Whether EMT is involved in cancer metastasis has a specific, practical impact on the field of circulating tumor cells (CTCs). Since the generally accepted definition of a CTC includes the expression of epithelial surface markers, such as EpCAM, if a cancer cell loses its epithelial surface markers (which is suggested in EMT), it will not be separated and/or identified as a CTC. We have developed, and previously reported on the use of, a purely negative enrichment technology enriching for CTCs in the blood of squamous cell carcinoma of the head and neck (SCCHN). This methodology does not depend on the expression of surface epithelial markers. Using this technology, our initial data on SCCHN patient blood indicates that the presence of CTCs correlates with worse disease-free survival. Since our enrichment is not dependent on epithelial markers, we have initiated investigation of the presence of mesenchymal markers in these CTC cells to include analysis of: vimentin, epidermal growth factor receptor, N-cadherin, and CD44. With the aid of confocal microscopy, we have demonstrated not only presumed CTCs that express and/or contain: a nucleus, cytokeratins, vimentin, and either EGFR, CD44, or N-cadherin, but also cells that contain all of the aforementioned proteins except cytokeratins, suggesting that the cells have undergone the EMT process. We suggest that our negative depletion enrichment methodology provides a more objective approach in identifying and evaluating CTCs, as opposed to positive selection approaches, as it is not subjective to a selection bias and can be tailored to accommodate a variety of cytoplasmic and surface markers which can be evaluated to identify a multitude of phenotypic patterns within CTCs from individual patients, including so-called EMT as presented here.

## Introduction

Based on the NCI-SEER data, an estimated 52,610 men and women will be diagnosed with squamous cell carcinoma of the oral cavity, pharynx, and larynx in 2012, accounting for 11,500 deaths. The majority of these head and neck cancers will be of squamous cell origin and arise from the mucosal lining [1], [2].

Squamous cell carcinomas of the head and neck (SCCHN) are associated with a relatively poor prognosis. For all stages of presentation, the 5-year survival rate is approximately 50% [3], and this mortality rate has not changed significantly in the last several decades despite advances in our understanding of disease biology and improvements in chemotherapy. Even with all the technological advances made in the molecular analyses of tumors, including the ability to sequence entire tumor genomes, the presence of regional nodal involvement is still the single most important prognostic marker for SCCHN. Often times, the presence of nodal disease is clinically occult in SCCHN and thus surgery is recommended to exclude microscopic nodal involvement. For instance, in SCCHNs originating from the oral cavity, approximately 25 percent of patients are demonstrated to have nodal disease microscopically despite no clinical evidence of disease [3]–[5]. Therefore, in order to properly stage many SCCHNs, surgical dissection is performed to sample regional lymph nodes.

In order for SCCHN to spread from its primary location, i.e. the oropharynx, to regional lymph nodes (other than by direct extension) the tumor must gain access to the lymphvascular channels. Once these cells detach from their primary tumor and enter the peripheral circulation they are officially designated circulating tumor cells (CTCs) and are able to deposit within lymph nodes and other organs where they may proliferate and develop into eventual metastatic tumors. The conventional definition of a CTC is an epithelial derived, nucleated, often cytokeratin-positive cell that is not a normal constituent of blood, and it is generally assumed that CTCs should not be identified in patients without an epithelial malignancy [6]. Therefore, the ability to detect CTCs in SCCHN patients might potentially serve a number of clinically useful purposes, including: 1) as a surrogate marker for nodal/metastatic disease which could potentially limit elective neck dissection surgery for staging in these patients; 2) as a marker for assessing treatment susceptibility; and 3) as a marker for post-treatment cancer surveillance.

Despite the potential clinical utility behind assessing for the presence of CTCs, we believe that the ability to definitively phenotype and genotype these CTCs could also provide detailed insight into the metastatic process behind SCCHN, as well as other carcinomas, and permit direct exploration of targeted treatment strategies [7], [8]. These concepts have led to the belief that CTCs could potentially serve as “liquid biopsies” in the future. However, there are significant challenges to overcome before the concept of the “liquid biopsy” may ever be realized, which include both physiological and technological aspects [9].

There is a growing body of literature that suggests that cancer originating from cells of epithelial origin undergo a process called *epithelial-mesenchymal transition* (EMT) in which the tumor cells undergo a loss of polarity, lose cell-cell junctions, and acquire a mesenchymal phenotype [10], [11]. It is also suggested that this mesenchymal phenotype is motile, migratory and invasive and that these properties promote the escape of the cancer cells from the primary site and aid in the development of metastases [10]–[14]. However, this concept is intensely debated; for example, why so-called EMT is observed in cell lines and animal models, and not definitively in vivo [15]–[17].

**Figure 1:**
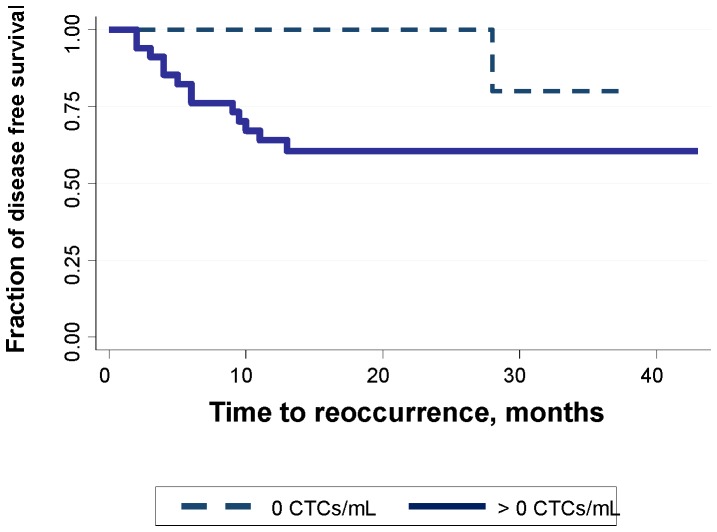
Kaplan-Meier Disease-Free Survival Plot. This KM plot, with respect to the number of CTCs per mL of SCCHN patient blood at time of surgery, is updated (with respect to time, no new patient samples) since previously published by Jatana *et al.* 2010. The Log-rank Pr>chi2 = 0.0335.

Given our initial results suggesting the potential of CTCs to have prognostic significance in SCCHN patients using our purely negative depletion technology (Figure 1), we began investigating differentiating markers, including those associated with a mesenchymal phenotype and cancer “stem cells”, on these enriched cells in order to gain potential insight into the metastatic process [18]–[22]. A number of reports indicate that a hallmark of cells undergoing EMT is a shift in cytoplasmic proteins from keratins to vimentin, and a shift in cell surface markers from E-cadherin to N-cadherin [23]–[25]. Further, with respect to SCCHN, reports suggest that upwards of 90% of primary tumors also overexpress EGFR, a protein not typically observed on blood cells, which might also aid as a diagnostic marker for this cancer type. We previously demonstrated that we could conduct confocal analysis upon these potential CTCs from SCCHN patients and demonstrate the localization of cytokeratin staining to the cell's cytoplasm [20]. Consequently, in this report we have extended this confocal analysis to other cell surface and cytoplasmic markers that we hypothesize can be associated with CTCs. These include the cytoplasmic protein vimentin, and three different cell surface proteins; EGFR, N-cadherin, and the reported cancer stem cell maker, CD44.

Herein, we highlight important findings realized from the evaluation of CTCs from SCCHN patients using our negative enrichment methodology. These findings include data which suggests that so-called EMT does occur in CTCs from SCCHN patients and in order to thoroughly evaluate a patient's blood for CTCs a methodology must be employed which is 1) not prone to selection bias, 2) tailorable and able to evaluate a multitude of cytoplasmic and surface proteins, and 3) most importantly utilizes positive and negative controls which aid in demonstrating the specificity of the employed staining protocol.

## Materials and Methods

### SCCHN blood collection

4.5 to 18.5 mls of peripheral blood was obtained from patients with a diagnosis of SCCHN undergoing surgical resection for their disease and whom had not been previously treated for this disease. The procedure was approved by the **Cancer Institutional Review Board of The Ohio State University Medical Center** (2004C0096), initiated on March, 2007 and informed, written consent, was obtained from all subjects. Blood samples were collected from a venous line, either immediately prior to, and/or post, surgery in BD-Vacutainer tubes, (BD Biosciences, Cat#366480) and stored at 4°C until processing. Samples were processed within 24 hrs after procurement. Operators were blinded to clinical correlative information during the blood sample processing and analysis.

### “Normal control” blood collection

11 to 18 ml of peripheral blood was obtained from adult women and men presenting for evaluation at the Walter Reed Army Medical Center Comprehensive Breast Care Center. These subjects subsequently underwent a diagnostic or therapeutic procedure for suspected breast cancer and did not have a prior history of invasive carcinoma or clinically-apparent metastatic disease at the time of the procedure. The study samples were drawn under was approved by the Cancer Institutional Review Board of the Walter Reed Army Medical Center (IRBnet #354344), initiated in January 2011 and informed, written consent, was obtained from all donors. Peripheral venous blood was collected in BD-Vacutainer tubes (Sodium Heparin), (BD Biosciences) and shipped overnight to The Ohio State University for immediate processing. Accurate records were taken, and all samples were processed within 30 hours of blood draw. Operators were blinded to clinical correlative information during the blood sample processing and analysis. None of the “normal control” blood sample donors were subsequently shown to have pre-invasive or invasive carcinoma.

### Cell culture and Cytospin procedure

Two breast cancer cell lines, MCF-7 and MDA-MB-231, and a head and neck cancer cell line, SCC-4,were procured from ATCC (Manassas, VA) and grown to mid-log phase in ATCC specified culture media, Dulbecco's Modified Eagle Medium (DMEM) (Cat#30-2002, ATCC, Manassas, VA), supplemented with 10% fetal bovine serum (FBS) (Cat#30-2020, ATCC, Manassas, VA) and 1% Penicillin-Streptomycin (Cat#30-2300,ATCC, Manassas, VA) at 37°C in 75 cm^2^ tissue culture flasks. SCC-4 cells were harvested by washing the adherent cells three times with PBS and subsequently incubating with Accutase™ (Cat#AT104, Innovative Cell Technologies, Inc.) for 5 min at 37°C to remove the attached cells from the T-flask. The Accutase™ was then neutralized with the culture media before pelleting the cells at 350×g for 5 min. The two breast cancer cell lines, MCF-7 and MDA-MB-231 werecultured and prepared in the same manner as the SCC-4 cell line.

The microscopic slides used for analysis (previously coated with poly-lysine) were first wetted by applying 200–250 µL of phosphate buffered saline (PBS, pH 7.4), supplemented with 1% bovine serum albumin (BSA), onto the slides by centrifugation at 800 g for 5 min. The cell suspension (200 µL) was then added to the cytology funnel and spun at 800 rpm for 5 min. The slides were then air dried and fixed with 1% formaldehyde for 15 min at room temperature, washed with PBS, and stored at 4°C until use.

### Reagents used for cell separation

Tetrameric antibody complexes (TACs) from Stem Cell technologies (Cat.# 18259,Vancouver, BC) were used to immunomagnetically label peripheral blood leukocytes (PBLs) as previously presented [19]. The specific TACs used in this study were a pan-leukocyte marker that targets different isoforms of the CD45 cell surface antigen and dextran coated magnetic nanoparticles.

### Separation Methodology

**Figure 2:**
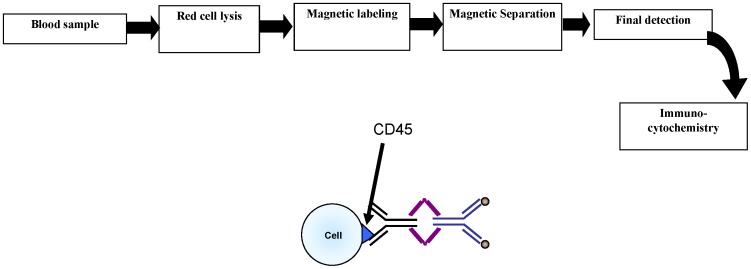
Schematic diagram of the CTC enrichment methodology.

The immunomagnetic separation was carried out as described in [19] and will only be briefly summarized here. An overall view of the separation process is shown in Figure 2. Red blood cells in the samples were lysed by mixing the sample with lysis buffer (154 mM NH_4_Cl, 10 mM KHCO_3_, 0.1 mM EDTA) at a ratio of 1∶25, incubating it for 5 min at room temperature, and then pelleting the remaining blood cells at 350× g for 5 min. This cell pellet, consisting mostly of nucleated cells, was then labeled with 0.5 microliter of anti-CD45 TAC per 1 million cells for 30 min at room temperature on a shaker. Without washing the cells, 1 microliter per 1 million cells of the magnetic nanoparticles were added to the cell suspension and incubated for 15 min at room temperature on a shaker. The immunomagnetically labeled cell suspension was subsequently diluted with 5 to 10 mls of buffer and run through the deposition Magnetic Sorter system to obtain an enriched sample.

The final cell suspension, which had passed through the magnetic sorter, was then measured for volume and the cell suspension was split into two aliquots; one of which was subsequently subjected to cell lysis and RNA preservation procedures and the other either directly cytospun for analysis or preserved with 10% neutral buffered formalin for later concentration and ICC analysis. Final cell numbers were obtained by diluting 10 microliters of the enriched cell suspension in 3% acetic acid (1∶10 dilution) and counting the number of total cells present using a hemocytometer.

### Immunostaining

Previously made cytospins of cells (described above) were hydrated in PBS for 5 min at room temperature (RT). Subsequently, the cells were permeablized with 0.25% Triton X-100 (T-100) in PBS for 15 min at RT and then blocked with 1% BSA in PBS for 1 hr at RT. The SCCHN cell line SCC4, was used to develop the labeling protocols and act as positive controls for all immunofluorescent stains employed in this study, except for EpCAM which is not expressed on this cell line. MCF-7 and MDA-MB-231 cell lines were utilized to develop the labeling protocol for EpCAM and act as a positive control. Table 1 summarizes all antibody reagents used in the immunofluorescence studies. Since it was not possible to do single step antibody labeling for all of the different markers, (i.e. targeted clones conjugated to specific fluoroprobes were initially not available) in some cases a single step labeling was used, and in other cases a two-step labeling was used. First, primary antibodies against vimentin and the surface markers CD44, EGFR or N-cadherin were diluted in an antibody diluent (Cat.#S3022, DAKO) and then the slides were incubated within the antibodies cocktail for 1 hr at RT. Subsequently, one of the appropriate secondary antibodies, AlexaFluor® conjugated IgGs, were applied for 1–2 hr at RT. The cells were once again blocked with 1% BSA in PBS for 30 min at RT, prior to incubating with FITC conjugated anti-cytokeratin antibodies, diluted in 0.1% T-100 in PBS, for 30 min at 37°C. The slides were washed with PBS for 15 min (5 min, 3 times) in between each labeling step and finally air dried and mounted with Vectashield® mounting medium with 4′,6-diamidino-2-phenylindole (DAPI) (Cat# H-1200, Vector Laboratories).

**Table 1:** Antibodies and fluoroprobes used in this study.

Target	Antibody clone	Manufacturer/Cat #	Fluoroprobe	Secondary antibody	Fluoroprobe
Nucleus	----		DAPI	----	-----
8, 18, 19 cytokeratins	CK3-6H5	Miltenyi Biotec 130-080-101	FITC	----	-----
8, 18, 19 cytokeratins	CK3-6H5	Miltenyi Biotec 130-080-101	AF488		
CD45	H130	BD Pharmingen 555480	AF594, custom conjugated		
EpCAM	Polyclonal, rabbit	AB71916	----	Anti-Rabbit	AF647
Vimentin	C-20, goat or polyclonal, goat	Santa Cruz Biotechnology SC-7557 or Abcam ab11256	----	Rabbit, anti-goat	AF555
				Rabbit, anti-goat	AF647
EGFR	polyclonal, rabbit	Abcam ab2430	----	Donkey, anti-rabbit	AF633
CD44	G44-26, mouse	BD Pharmingen 559942	APC	-----	----
N-cadherin	polyclonal, rabbit	Abcam ab12221	----	Donkey, anti-rabbit	AF633

Given the time period over which the data in this publication has been obtained, two distinct staining protocols and antibody fluoroprobe conjugates have been utilized. The initial staining protocol included: DAPI, anti-cytokeratin-FITC, anti-vimentin (with a secondary fluorprobe conjugated antibody targeting the primary anti-vimentin antibody), and the fourth antibody targeting either EGFR, CD44, or N-Cadherin (with a secondary conjugated antibody targeting the primary antibody) (Table 1). The second, improved, labeling protocol used: DAPI, anti-cytokeratin antibody directly conjugated to AF488, anti-CD45 directly conjugated to AF594, and the fourth marker (EpCAM or vimentin) using a two-step labeling. While aliquots of enriched patient blood were preserved from the samples used to produce the Kaplan Meier plots in our previous publication (Figure 1), optimal staining results are obtained from samples less than three months old. Consequently, five new peripheral blood samples from SCCHN patients meeting the original criteria defined above were obtained for staining studies using these two new protocols. Microscopic observations of the cytospins indicated that three of the five enriched blood samples had cytokeratin positive cells (CTCs). This ratio is consistent with what was reported by Jatana et al. (2010) [21].

### Microscopic Analysis

Once both labeling protocols had been established, a subset of the SCCHN patient samples that had previously demonstrated the presence of CTCs were chosen for further analysis using confocal microscopes. Three microscopes was used in this study, an epifluorescence Nikon microscope and two confocal systems: a Zeiss LSM 510 confocal/multi photon laser scanning microscope (Carl Zeiss MicroImaging Inc.) and an Olympus FluoView1000 spectral confocal system (Olympus Inc.). The Zeiss LSM 510 confocal/multi photon laser scanning microscope was equipped with HeNe1 (543 nm), HeNe2 (633 nm) laser, Argon/2 laser (458,477,488,514 nm) and a Titanium Sapphire laser (750 nm). The cells were viewed with a 63× (NA 1.2) apochromatic water objective and images of different fields were taken. The argon, HeNe1, and HeNe2 lasers, with emission filters of BP 500–550 nm, LP 560, and LP 650, were used for visualization of FITC, AF 555 or 594, and APC (or AF633), respectively. The DAPI staining was viewed by the use of a tunable IR laser which excites the fluorophore using two photons at the point of focus and a BP of 435–485 nm.

The Olympus FluoView1000 laser scanning microscope, using FVIOASW software, was equipped with HeNe1 (543 nm), HeNe2 (633 nm), Argon/2 laser (458, 477, 488, 514 nm) and a 405 nm diode laser for DAPI. The cells were viewed with a 60× oil objective and images of different fields were taken. For this specific study, the 405 diode, argon, HeNe1, and HeNe2 lasers, were used with the software selected emission filters for the DAPI, FITC, Alexa Fluor 555 or 594 and APC or AF633, respectively.

### Method of determining number of cells per slide

The slides were scanned in a single field of view increment (20× objective, Nikon microscope) and cells that were positive for FITC, and subsequently AF488 and DAPI (cytokeratin positive with a nucleus) were counted as a CTC. After scanning the slide along the horizontal axis once, the objective was moved vertically by one field of view increment, and the slide was scanned again for double positive events. This process was repeated until the entire area of the cell spot was covered. The total number of CTCs were calculated as:


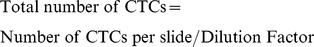


## Results

Table 2 lists the patient demographics, pathological stage, number of CTCs/ml determined (using the criteria reported above), and outcomes for analyzed samples. It also provides a count of the number of DAPI+, CK+ cells per ml of peripheral blood, as well as the number of DAPI+, CK−, vimentin+, and either EGFR, CD44, or N-cadherin+cells per ml of peripheral blood.

**Table 2:** Patient data.

Intra-operative Patient Blood Sample*	Volume of blood collected (mls)	Age/Gender	Location	General Site	Path Stage	Overall Stage	Nodal involve-ment	Extra-capsular Spread	Perineural spread	Vascular invasion	Dif-ferentiation	Outcome	# CK+, Vimentin+, Either EGFR, C44, or N-cadherin+/ml of blood	# CK−, Vimentin+, Either EGFR, C44, or N-cadherin+/ml of blood
1 post	15	65M	Gingiva	OC	T4aN0M0	4	0/30	N/A	Absent	Absent	Well	4 m R	3	24
2 post	10.5	56F	BOT	OP	T3N0M0	3	0/1	N/A	Present	Present	Moderate	2 m R	65	176
3 post	11.2	44M	Tonsil	OP	T2N2bM0	4	4/55	Absent	Absent	Absent	Moderate	31 m NED	149	170
4 post	10.2	26F	Tongue	OC	T2N0M0	2	0/22	N/A	Present	Absent	Moderate	2 m R	214	96
5 post	5.5	55M	Tonsil	OP	T2N0M0	2	0/52	N/A	Absent	Absent	Well	39 m NED	748	391
														
6 pre	10	70F	BOT	OP	T3N2cM0	4	8/30	Present	N/A	N/A	Moderate	4 m R	2171	828
6 post	11.6												3307	3969
7 pre	9.1	57F	Oral	OC	T4N0M0	4	0/32	N/A	Present	Absent	Moderate	36 m NED	243	0
7 post	9												214	0
8 pre	15.4	56M	BOT	OP	T3N2cM0	4	34/43	Present	Present	Present	Moderate	9 m R	915	229
8 post	10.7												2260	0

* Pre corresponds to sample taken immediately prior to surgical intervention in the OR; Post corresponds to sample taken post- surgical intervention, but still within the OR.

Differentiation.

### Four color staining of cells

**Figure 3:**
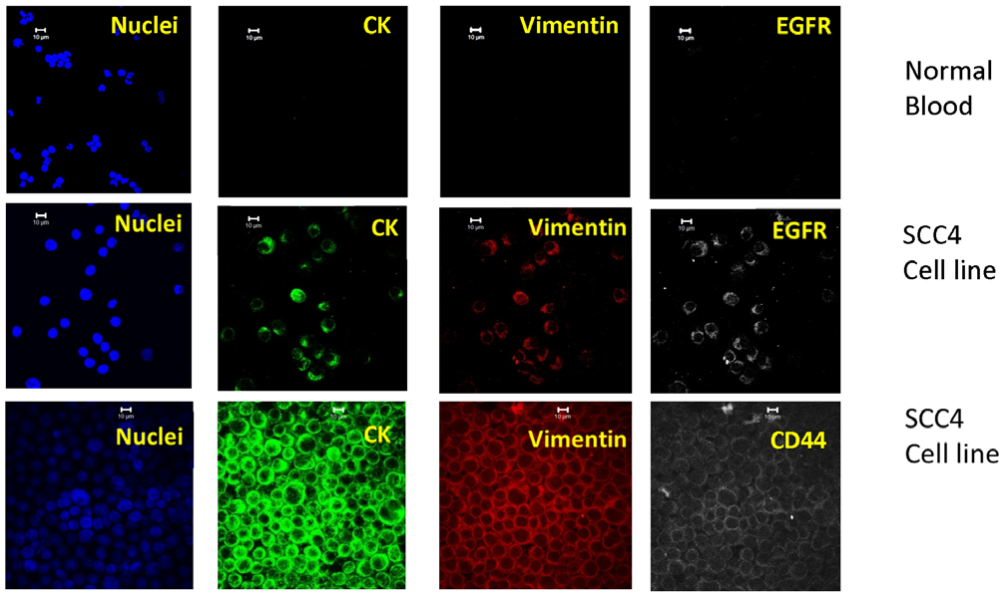
Representative set of confocal images of a cytospin of stained normal blood samples and SSC4 cell lines. The cell structure/protein targeted is listed in each image, the type of cells listed on right. Note, the second row of images presents cells stained for EGFR, while the third row shows cells stained for CD44.

Figure 3 is a representative set of confocal images (Zeiss) of normal blood and SCC4 cytospins stained with the initial staining protocol of DAPI, cytokeratins, vimentin, and EGFR or CD44. No staining beyond DAPI was observed in “normal control” human blood (n = 10).

**Figure 4:**
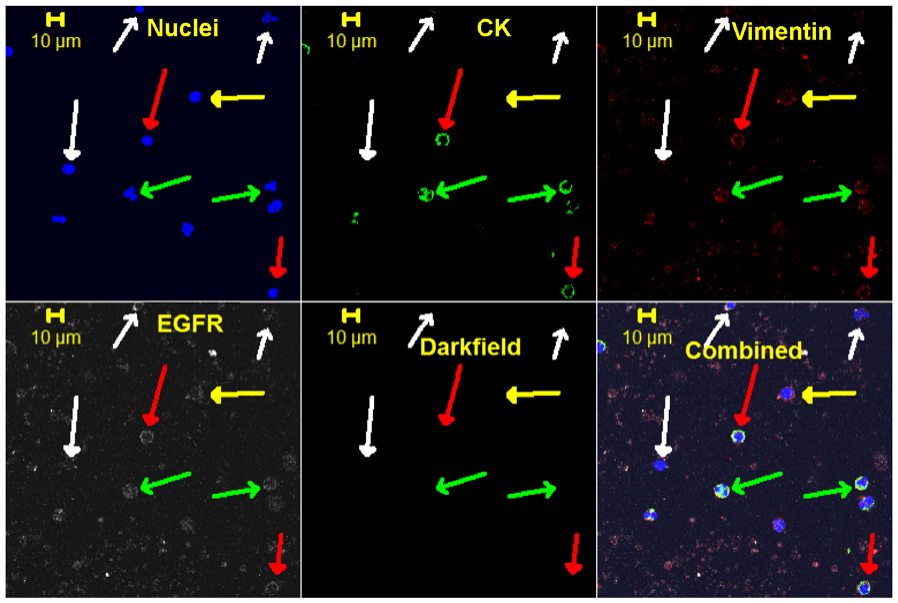
Representative, confocal images of a cytospin of an enriched peripheral blood sample from a 27 year old, HPV positive, SCCHN patient. Sample taken in the OR, 214 cytokeratin positive cells per ml of blood were detected, and she had a reoccurrence 2 months after surgery. For this specific four color staining, the cell structure/protein targeted is listed in each image. The cells highlighted with a red arrow are positive for all four markers, yellow are negative for CK, weak or negative for vimentin and EGFR, and white is negative for all but nuclei. Green arrows highlight cells that have a granulocytic like nuclei.

**Figure 5:**
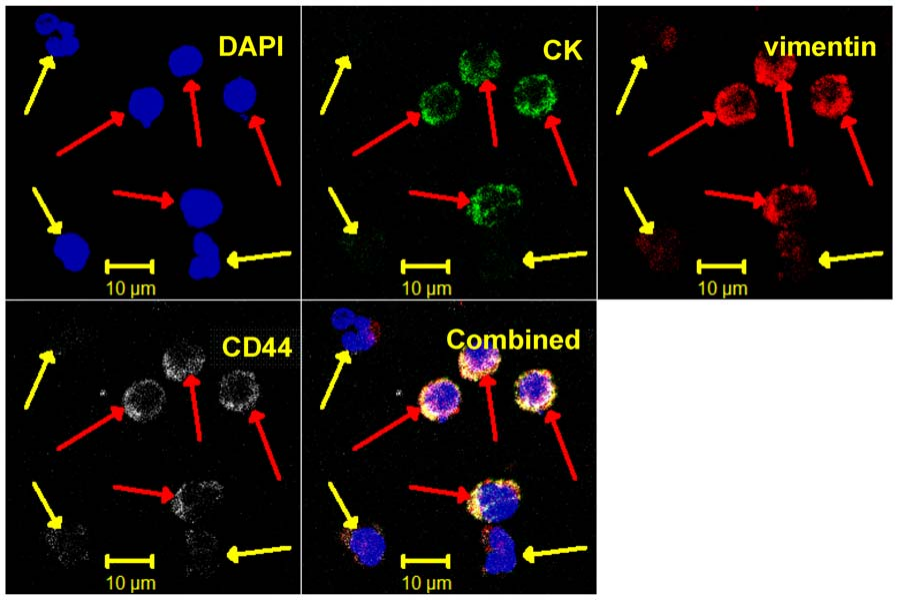
Representative, confocal images of a cytospin of an enriched peripheral blood sample from a SCCHN patient. This is the same patient sample used to create the cytospin presented in Figure 4. However, for this specific, four color staining, the anti-EGFR antibody was replaced with anti-CD44. The cells highlighted with a red arrow are positive for all four markers, while cells highlighted with yellow arrows are either negative, or very weak for CK, vimentin, and CD44.

**Figure 6:**
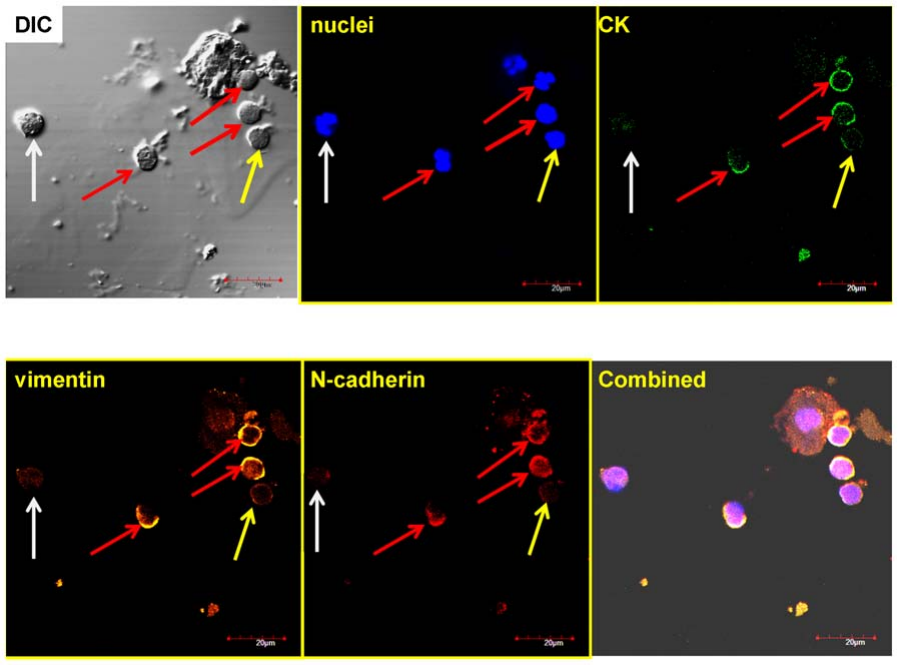
Representative, confocal images of a cytospin of an enriched peripheral blood sample from a 65-year-old male with Stage 4 SCCHN. The first image in the upper left is a DIC image which is an option on the Olympus confocal system. The cell structure/protein targeted is listed in each image. The cells highlighted with a red arrow are positive for all four markers, yellow are negative/weak for CK, vimentin and N-cadherin, and white is negative for all but nuclei.

**Figure 7:**
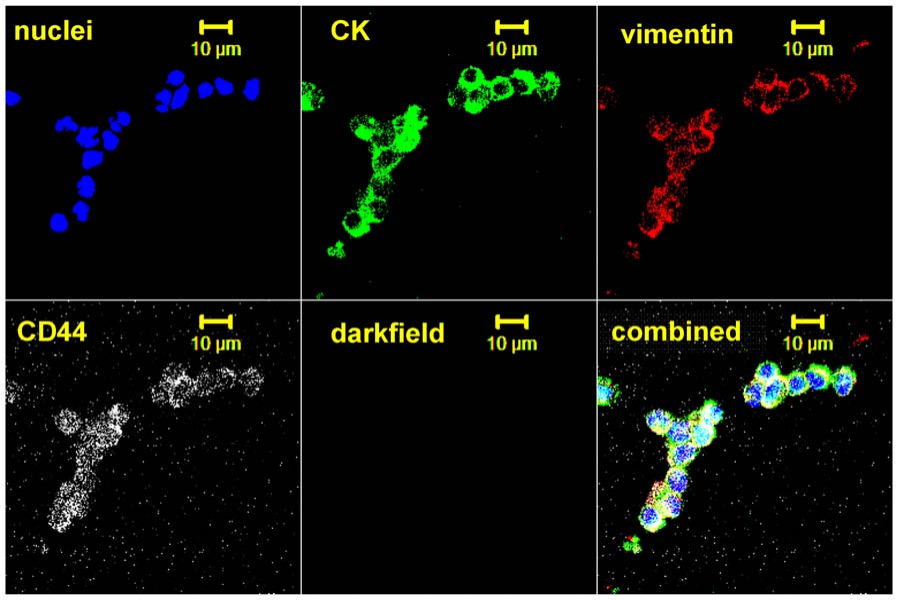
Set of confocal images of cohesive clusters of cell suggestive of tumor embolus from a 56-year-old male whom had a reoccurrence after 9 month. The cell structure/protein targeted is listed in each image. Note the range of staining intensity cytokeratins, vimentin, and CD44 within the cluster.

Figures 4, 5, 6, 7 are representative images of the initial staining protocol on enriched CTC samples of SCCHN patients. Figures 4 and 5 were from a 27-year-old,female with Stage 2, HPV-positive SCCHN who presented with 214 cytokeratin positive cells per ml of blood at the time of sampling. Her tumor recurred 2 months after surgery. Figure 6 represents blood from a 65-year-old male with Stage 4 SCCHN, and Figure 7 is from a 56-year-old male also with Stage 4 SCCHN that recurred 9 months after the sample was obtained.

In each set of images, the first three colors represent: 1) DAPI, 2) CK, and 3) vimentin, while the fourth color is either CD44 or N-cadherin, respectively. Figures 3, 4, 5, & 7 utilize the Zeiss confocal microscope, while Figure 6 utilizes the Olympus confocal microscope, which allows for the use of differential interference contrast (DIC) imaging. Figure 4 and 5, while stained for a different fourth color, come from the same enriched patient sample and suggest that the cells present express all four markers in both, implyingthat the identified cells expressing CD44 are also probably positive for EGFR. In addition, close evaluation of Figures 4 and 5 demonstrate differential expressions of marker staining. Without further fluorescent signal quantification, such conclusions are only speculative, but the images appear to demonstrate that vimentin-positive/CK-negative cells seem to express much lower levels of vimentin as compared to vimentin-positive/CK-positive cells. The significance of this is unknown. A similar range in signal intensity can be observed in Figure 7.

### Four color staining and confocal analysis including EpCAM and CD45

**Figure 8:**
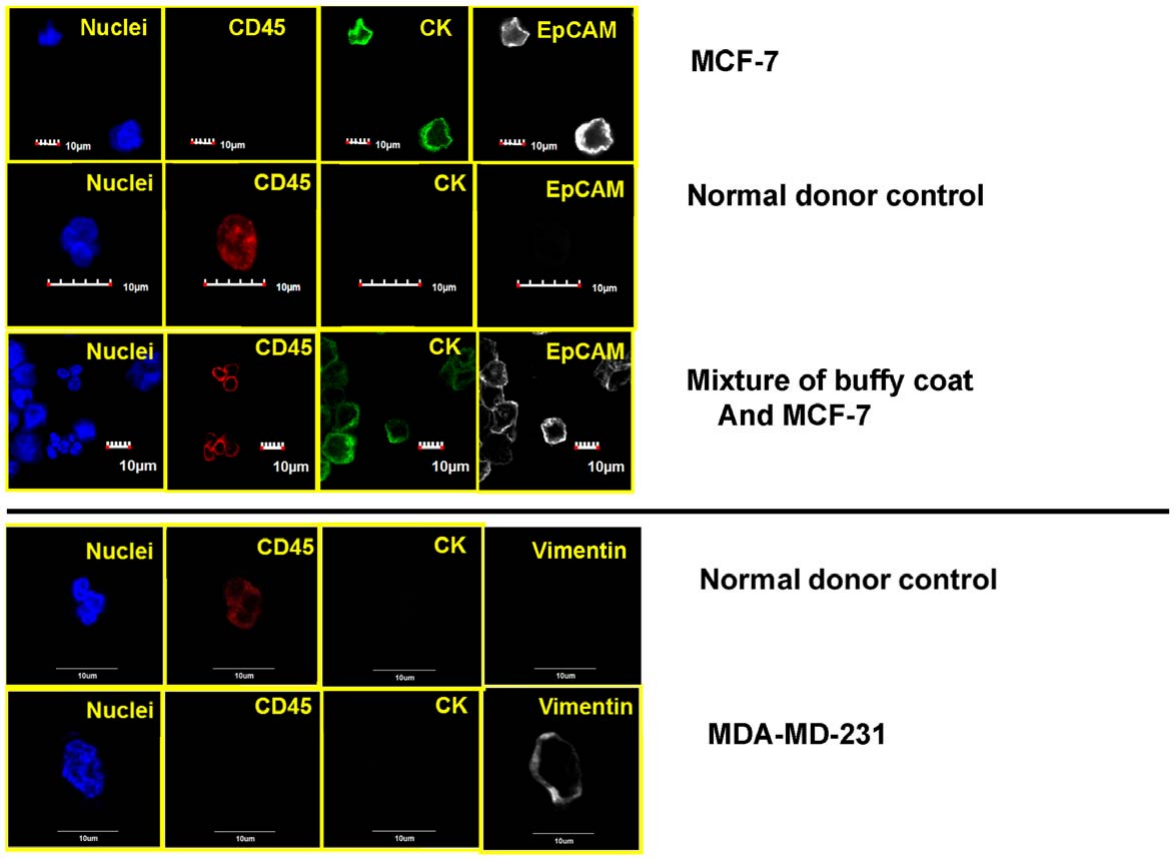
Representative set of confocal images of a cytospin of normal stained human blood and two breast cancer cell lines. The cell structure/protein targeted is listed in each image, the type of cells listed on right.

**Figure 9:**
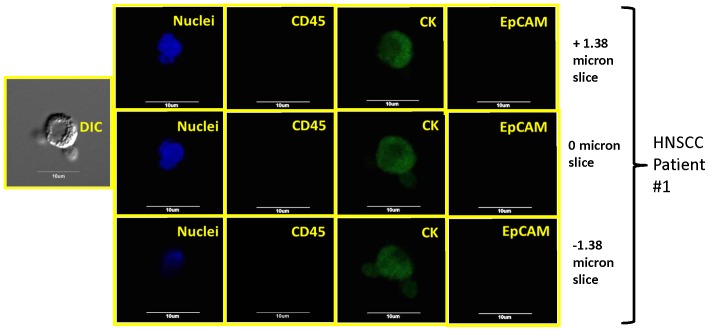
Representative, confocal images of an enriched peripheral blood samples from a SCCHN patient. Each row of images correspond to a different, confocal, z-slice: 1.38 microns above the middle plane, the middle plane (relative to cell center), and 1.38 microns below the middle plane. A total of 18 z- slices were made, and no indication of CD45 or EpCAM presence was observed in any of the slices. For this set of images, custom conjugated anti-cytokeratin AF 488, anti-CD45 AF594, and a two set, rabbit, anti-EpCAM and anti-rabbit, AF647 was used for staining.

To determine the CD45 status of the enriched blood samples, the second labeling protocol was developed. Figure 8 represents positive and negative controls for this protocol. This protocol was performed on more recent SCCHN blood samples, and Figure 9 represents confocal images from one of these samples containing CK-positive/CD45-negative cells. The three rows of images are representative “z slices” (of a total of 18 slices taken) of a higher magnification (relative to previous images presented) of a single cell. In none of the other non-presented “z-slices” was any indication of CD45 or EpCAM expression identified.

**Figure 10:**
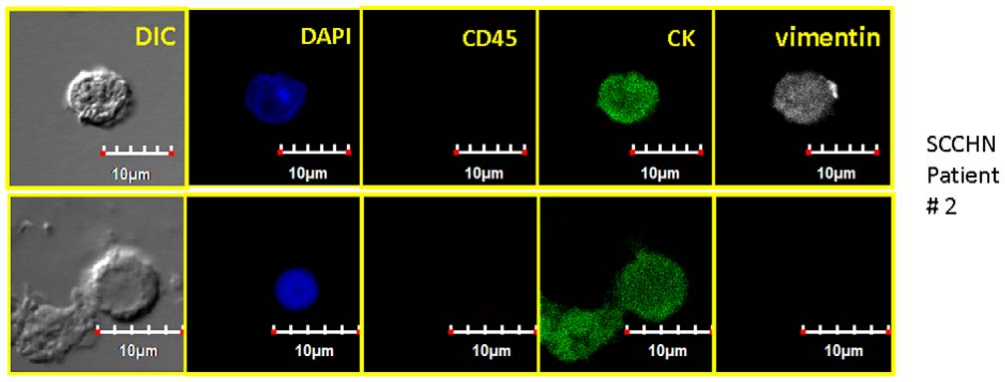
Representative, confocal images of an enriched peripheral blood samples from a HNSCC patient. Both rows of images are from the same patient, different cytospin locations. These two sets of images illustrate the presence and absence of cells negative for CD45, yet positive for both CK and vimentin as well as only positive for CK. Also presented is a “ghost” of what appears to be a cell which has lost its nuclei.

Figure 10 represents two different image sets from one of the three more recent SCCHN patients in which the anti-EpCAM antibody was replaced with an anti-vimentin antibody. As with Figure 9, a full set of z-slices were made, but only the slice cutting through the middle of the cell is presented.

## Discussion

We suggest that this study presents a number of significant findings. Using specific, custom, antibody fluoroprobe conjugates, we enriched for, and phenotyped, putative CTCs from the peripheral blood of SCCHN patients and these enriched cell suspensions: 1) demonstrated a multitude of phenotypic patterns within individual patients, 2) demonstrated phenotypes suggestive of so-called EMT, 3) highlighted that our enrichment and staining protocols can be tailored to a variety of markers, including demonstration of CD45 negativity, and 4) proved that our negative enrichment approach allows us to isolate putative CTCs that are negative for epithelial makers, such as EpCAM, and is therefore not subject to a positive selection bias. Additionally, through the evaluation of “normal control” blood samples and the control cell lines, we were able to demonstrate that our evaluation of these CTCs is not significantly hampered by non-specific antigen uptake/binding.

Several methodologies have been developed to detect and characterize CTCs in peripheral blood of cancer patients. These methodologies fall under three main categories: immunological based assays, molecular based assays, and physical based, such as filtration. Although these approaches can be used directly, an enrichment step prior to the detection is usually preferred. A number of methodologies exist for enriching for rare cancer cells, including density gradient separation to enrich for nucleated cells (i.e., Ficoll gradient separation), and magnetic cell separation either targeting the cancer cell, positive immunomagnetic cell separation (PIMS), or targeting normal blood cells, negative immunomagnetic cell separation (NIMS). Unfortunately, most of the reported studies using some form of magnetic cell separation to separate or enrich rare cancer cells do not provide data, or complete data, on the performance of the magnetic separation step. Consequently, it makes quantitative comparison of the performance of technology, as well as the overall study, difficult or impossible to evaluate.

Common PIMS approaches to recover CTCs suffer from some degree of selection bias since they assume that all CTCs will express the epithelial surface marker EpCAM or some other common cytokeratin filament [9]. Additionally, these approaches could also miss CTCs which are potentially cloaked with platelets, as well as missCTCs that do not express the targeted epithelial markers; for example, CTCs that have gone through a presupposed epithelial to mesenchymal transition [10].

In order to mitigate this selection bias, a negative depletion separation approach can be taken in which only normal cells are removed; leaving behind, ideally, only abnormal unlabeled cells. After using this approach, the remaining cell suspension is then further analyzed to positively identify a cell as a CTC. This method of analysis of the enriched cell product is significantly more flexible since no assumption is made with respect to a CTC in the enrichment stage (except that it is negative for CD45). A number of negative depletion approaches have been reported, including the removal of red blood cells and the removal of specific white blood cells [23]–[25]. Unfortunately, many of these studies do not report the effectiveness of the process; i.e. the number of logs of depletion of the targeted cell, and when spiking studies are conducted, the recovery of the spiked cells.

In contrast, we have been developing, and reporting the performance, of both positive selection and negative enrichment, magnetic cell separation technologies for a number of cell types and applications for a number of years [18]–[22]. With respect to the enrichment of circulating tumor cells, we have taken a similar approach as others in which we perform a red blood cell lysis step, followed by immunomagnetically targeting CD45 positive cells, in a specifically designed system. As with T-cell depletion studies, spiking tests with some, but not all, cancer cell lines we are able to recover greater than 80 percent of the spiked, cultured tumor cells [18], [19].

Given this level of recovery of spiked cancer cell lines, we initiated a study using this optimized technology for CTC enrichment, and subsequent CTC enumeration based on the presence of nuclei and stained cytokeratin proteins, Jatana et al. (2010). Figure 1 is an updated Kaplan-Meier disease-free survival plot (updated with respect to time since initial data taken; no new patients) from this publication by Jatana et al. demonstrating the potential of CTCs as a prognostic marker of disease-free survival in this disease entity.

Consistent with this initial study, this current study indicates the presence of cells in the peripheral blood of patients with SCCHN that contain not only cytokeratins, but also express vimentin and N-cadherin suggesting that these cells potentially have undergone an EMT. While there are reports of the detection of molecular markers for mesenchymal markers (i.e. PCR products) in the blood of mesenchymal markers we are not aware of any other reports that present multiparameter, immunochemical staining of enriched cells in the blood of cancer patients. We suggest that the subpopulation of cells that are weak for cytokeratins, but positive for the other three markers, are cells which may potentially be undergoing so-called EMT, and that these cells should also be classified as CTCs as they are most likely associated with the patient's SCCHN.

The reliance of a single marker to define a specific cell type introduces significant potential for misclassification and can be applied broadly to any marker. We were able to demonstrate this point with our analysis of EpCAM on the enriched CTCs identified within this study (Figures 9 and 10). That said, often times some markers are so important that evaluation must be performed in order to maintain some level of thoroughness. In our initial study evaluating CTCs in SCCHN [21], we classified a cell to be a CTC based on DAPI and cytokeratin 8, 18, 19 expression (visual detection of dye), and indirectly assuming that the cells were not positive for CD45 based on magnetic depletion of these cells. In this report, under the first staining protocol four markers (DAPI, cytokeratin, vimentin, and EGFR, CD44, or N-cadherin) were used to further evaluate the cells initially defined as CTCs and associated with the patients SCCHN. We have not observed, nor are we aware of any reports, that suggest that these three or four markers, especially in a combined manner, are expressed on normal blood cells. We subsequently confirmed this with multiple control studies on blood from “normal” patients. Moreover, in order to address our shared concern regarding the lack of definitive CD45-negativity on the cells of interest, the second set of staining protocols was developed which included both CD45 and either EpCAM or vimentin. We subsequently identified numerous examples of CK positive, CD45 negative cells. Interestingly, no cells were positive for EpCAM highlighting a potential fault of methodologies which utilize a positive selection relying upon this marker, at least for SCCHN patients.

A further, commonly discussed criterion for CTC is morphology and/or size; specifically that CTCs are typically larger than normal blood cells. As shown in the figures presented, all of the confocal images include a microscope/computer generated scale bar of either 10 or 20 microns. Consistent with commonly assumed values, the human cancer cell line SCC4 measures on the order of 20 microns, Figure 3, when collected on a cytospin slide. Conversely, normal human blood cells are, on average, 10 microns or less. However, as presented in Figures 5, 6, 7, 8, 9, 10, the cells that we are associating with the patient's cancer can be on the order of 10 microns or less. Given the relatively rapid recirculation of human blood, and relatively small capillary diameters, it is not surprising that many of the cancer associated cells we have identified demonstrate a size similar to that of normal human blood. Techniques to identify CTCs that depend on cellular size for identification may only select for a subpopulation of all of the cells of interest.

An argument can be made that these CTCs with the additional markers identified in this study could be the result of cells released from tissue/tumor during surgery. Table 2 lists 8 distinct SCCHN patients with identified CTCs which we conducted four color, confocal analysis upon. The first four patients listed only had blood samples taken in the operating room after surgical intervention, while the last four patients had samples taken prior to, and after surgical intervention, but while still in the operating room. These four paired samples are part of a larger study to determine if surgery releases CTCs into circulation. While too small of a sample size to draw any conclusion with respect to the effect that surgery has on the presence of CTCs, they do indicate that in all four patients, the results prior to and after surgical intervention are qualitative consistent.

We have conducted a multitude of three (DAPI, cytokeratin, and vimentin) and four color staining analyses on other patient's samples using the Nikon epifluorescence microscope which are not reported here. While all the cells that were DAPI and cytokeratin positive appeared to also be vimentin and either EGFR or CD44 positive, we are not as confident with respect to our ability to distinguish between background signal and positive signal in some of the samples using purely our eyes, or the typical imaging software that comes with the fluorescent microscope for signals above 700 nm (the fourth “color”). Consequently, despite the use of appropriate filters in the epifluorescence analysis we elected to not present that data. However, in order to address these limitations, we employed confocal microscopy and utilized custom conjugated antibodies with Alexa Fluor dyes (second staining protocol) that have a significantly smaller range in emission and are significantly more photostable. While accurate, confocal microscopy is labor intensive and expensive which precludes it use in the routine analysis of patient samples.

### Conclusion

We suggest that these initial results indicate that there are other cells of interest, beyond the traditional definition of a CTC, in the blood of some SCCHN cancer patients, and that these cells express markers that are consistent with cells that have a mesenchymal phenotype. While molecular analysis of blood has reported the presence of these markers in cancer patient blood, this is the first report we are aware of that shows that these cells can be microscopically detected in the blood of SCCHN patients. These findings have important implications in the methodology used to identify CTCs as other positive selection techniques, those targeting cells with only one specific cell surface marker, could fail to identify such abnormal cells in the blood. We also wish to suggest that accurate immunochemical staining for more than three colors, as was done in this study, requires either the use of confocal microscopy or more advanced multispectral analysis, which we are currently developing, to prevent misinterpretation of cellular autofluorescence or other light noise as a positive signal for the specific marker. Finally, it is suggested that future, well-designed, prospective studies be performed to determine if correlations can be made with respect to these mesenchymal-like cells and patient outcomes.
